# Artificial intelligence in stroke risk assessment and management via retinal imaging

**DOI:** 10.3389/fncom.2025.1490603

**Published:** 2025-02-17

**Authors:** Parsa Khalafi, Soroush Morsali, Sana Hamidi, Hamidreza Ashayeri, Navid Sobhi, Siamak Pedrammehr, Ali Jafarizadeh

**Affiliations:** ^1^School of Medicine, Tehran University of Medical Sciences, Tehran, Iran; ^2^Student Research Committee, Tabriz University of Medical Sciences, Tabriz, Iran; ^3^Tabriz USERN Office, Universal Scientific Education and Research Network (USERN), Tabriz, Iran; ^4^Neuroscience Research Center, Tabriz University of Medical Sciences, Tabriz, Iran; ^5^Nikookari Eye Center, Tabriz University of Medical Sciences, Tabriz, Iran; ^6^Faculty of Design, Tabriz Islamic Art University, Tabriz, Iran; ^7^Institute for Intelligent Systems Research and Innovation (IISRI), Deakin University, Geelong, VIC, Australia

**Keywords:** stroke, neurovascular disease, artificial intelligence, retinal images, fundus images, deep learning, machine learning, review

## Abstract

Retinal imaging, used for assessing stroke-related retinal changes, is a non-invasive and cost-effective method that can be enhanced by machine learning and deep learning algorithms, showing promise in early disease detection, severity grading, and prognostic evaluation in stroke patients. This review explores the role of artificial intelligence (AI) in stroke patient care, focusing on retinal imaging integration into clinical workflows. Retinal imaging has revealed several microvascular changes, including a decrease in the central retinal artery diameter and an increase in the central retinal vein diameter, both of which are associated with lacunar stroke and intracranial hemorrhage. Additionally, microvascular changes, such as arteriovenous nicking, increased vessel tortuosity, enhanced arteriolar light reflex, decreased retinal fractals, and thinning of retinal nerve fiber layer are also reported to be associated with higher stroke risk. AI models, such as Xception and EfficientNet, have demonstrated accuracy comparable to traditional stroke risk scoring systems in predicting stroke risk. For stroke diagnosis, models like Inception, ResNet, and VGG, alongside machine learning classifiers, have shown high efficacy in distinguishing stroke patients from healthy individuals using retinal imaging. Moreover, a random forest model effectively distinguished between ischemic and hemorrhagic stroke subtypes based on retinal features, showing superior predictive performance compared to traditional clinical characteristics. Additionally, a support vector machine model has achieved high classification accuracy in assessing pial collateral status. Despite this advancements, challenges such as the lack of standardized protocols for imaging modalities, hesitance in trusting AI-generated predictions, insufficient integration of retinal imaging data with electronic health records, the need for validation across diverse populations, and ethical and regulatory concerns persist. Future efforts must focus on validating AI models across diverse populations, ensuring algorithm transparency, and addressing ethical and regulatory issues to enable broader implementation. Overcoming these barriers will be essential for translating this technology into personalized stroke care and improving patient outcomes.

## 1 Introduction

Stroke is the second leading cause of mortality and the third most prevalent cause of disability globally, with 800,000 incidents annually in the US and a global mortality rate of approximately 5.5 million per year (Ovbiagele and Nguyen-Huynh, 2011; Roger et al., 2011). This condition arises from disrupted blood flow to the brain, either due to a blocked cerebral artery, leading to ischemic stroke (IS), which accounts for 87% of cases, or a ruptured artery, resulting in hemorrhagic stroke (HS), responsible for 10% of cases (Coupland et al., 2017). The prevalence of stroke is increasing, largely driven by demographic shifts such as extended life expectancy and the widespread adoption of high-risk lifestyle behaviors (Shafaat et al., 2022). Early prediction and prevention are crucial for mitigating this escalating global health challenge (Pandian et al., 2018).

The brain and retina share anatomical and physiological similarities due to their common embryological origin, both deriving from the neuroectoderm layer during embryonic development (London et al., 2013; Chua et al., 2021; Zhang Y. et al., 2022). These shared characteristics make the retina a valuable non-invasive tool for investigating the central nervous system (CNS) (Cheung et al., 2010). As a result, changes in retinal morphology, such as thinning of the retinal nerve fiber layer (RNFL) and retinal ganglion cell layer (RGCL), are strongly linked to neurological disorders like Alzheimer’s disease (Kesler et al., 2011; Cheung et al., 2014; Thomson et al., 2015; den Haan et al., 2017), Parkinson’s disease (Bodis-Wollner, 1990; Ortuño-Lizarán et al., 2018; Zhang Y. et al., 2022), and multiple sclerosis (Walter et al., 2012; Petzold et al., 2017; Pawlitzki et al., 2020). Stroke incidence and mortality have also been associated with retinal microvascular anomalies, including increased vessel tortuosity and arteriovenous nicking (Cheung et al., 2010; Cheung et al., 2017; Zhang Y. et al., 2022; Girach et al., 2024). These retinal changes can reflect the health of cerebral vessels and may serve as early indicators of stroke risk (Bodis-Wollner, 1990; Masugata et al., 2010; Kesler et al., 2011; Walter et al., 2012; Cheung et al., 2014; Thomson et al., 2015; Cheung et al., 2017; den Haan et al., 2017; Petzold et al., 2017; Ortuño-Lizarán et al., 2018; Pawlitzki et al., 2020; Girach et al., 2024). Furthermore, stroke risk factors such as hypertension (HTN), diabetes mellitus (DM), and atherosclerosis can adversely affect the retinal vasculature. These conditions often lead to hypertensive retinopathy, diabetic retinopathy (DR), and retinal vessel occlusions, which can serve as early indicators of CNS vascular diseases, including stroke (Klein et al., 2000; Masugata et al., 2010).

In order to stratify the risk of stroke occurrence in various patients, scoring checklists have been created, including Framingham Risk Score (FRS), CHA2DS2-VASc, ASCVD risk estimator, ATRIA, and Essen stroke risk scores. FRS combines the impact of age, sex, and baseline measurements of various vascular risk factors, including systolic blood pressure, the use of antihypertensive medications, the presence or absence of left ventricular hypertrophy on electrocardiography, pre-existing cardiovascular disease, current or previous atrial fibrillation (AF), current smoking status, and DM (Dufouil et al., 2017; Youssef et al., 2024). The CHA2DS2-VASc scoring system assigns 1 point to each condition: HTN, vascular disease, DM, age 65–74, and female sex. It awards 2 points for a history of stroke, transient ischemic attack (TIA), or thromboembolism and 2 points for age 75 and above. Nonetheless, none of these scoring systems consider specific organ changes, such as those in the retina, when calculating stroke risk (Olesen et al., 2012; Cetin et al., 2014; Jia et al., 2018). While neuroimaging provides direct evidence of brain health, it is often resource-intensive, expensive, and primarily used for diagnostic purposes rather than routine stroke risk prediction (Karthik et al., 2020). In contrast, retinal imaging offers a non-invasive, cost-effective, and accessible alternative, allowing for early detection of stroke risk through the assessment of retinal vascular changes that correlate with cerebrovascular health (Girach et al., 2024). Neuroimaging is critical for diagnosing established cerebrovascular conditions; however, retinal imaging can be used for ongoing preventive screening, especially in settings where advanced neuroimaging techniques are less accessible, making it an ideal tool for large-scale stroke risk assessment. Moreover, the modalities assessing the retina could be more advantageous if their outputs were combined with existing data on systemic changes induced by stroke risk factors and the latest neuroimaging techniques.

Retinal imaging presents a viable alternative to traditional neuroimaging methods like magnetic resonance imaging (MRI) and computed tomography (CT) for stroke risk assessment, primarily due to its non-invasive nature, accessibility, and cost-effective nature. Unlike MRI and CT scans, which require sophisticated infrastructure, retinal imaging can be performed with portable devices, making it more accessible, especially in resource-limited settings (Cheung et al., 2017; Pachade et al., 2022; Girach et al., 2024). Additionally, conducting frequent, and repeatable retinal assessments facilitates ongoing monitoring and early intervention, which are crucial for stroke prevention (Cheung et al., 2012; Pachade et al., 2022).

Advancements in artificial intelligence (AI) have opened new frontiers in medical imaging in recent years, particularly in stroke risk prediction and diagnosis (Daich Varela et al., 2023; Chakraborty et al., 2024). AI’s ability to process and analyze vast amounts of data allows it to uncover patterns that might be imperceptible to the human eye, making it a powerful tool for early disease detection. AI techniques have shown promise in enhancing the analysis of retinal images by improving image quality through processes like denoising, artifact reduction, precise segmentation, and classification of retinal features (Ali et al., 2020; Yedavalli et al., 2021). These advancements span a range of applications for stroke, from stroke risk stratification and diagnosis (Arbabshirani et al., 2018; Ni et al., 2018) to severity grading (Kogan et al., 2020; Park et al., 2020), personalized treatment planning (Quandt et al., 2023; Yao et al., 2023), prognosis (Bentley et al., 2014; Ramos et al., 2019; Zihni et al., 2020; Dengler et al., 2021; Lee et al., 2023), and evaluation of rehabilitation programs (Chae et al., 2020; Scrutinio et al., 2020; Campagnini et al., 2022; Guo et al., 2022; Zu et al., 2023).

**Figure 1:**
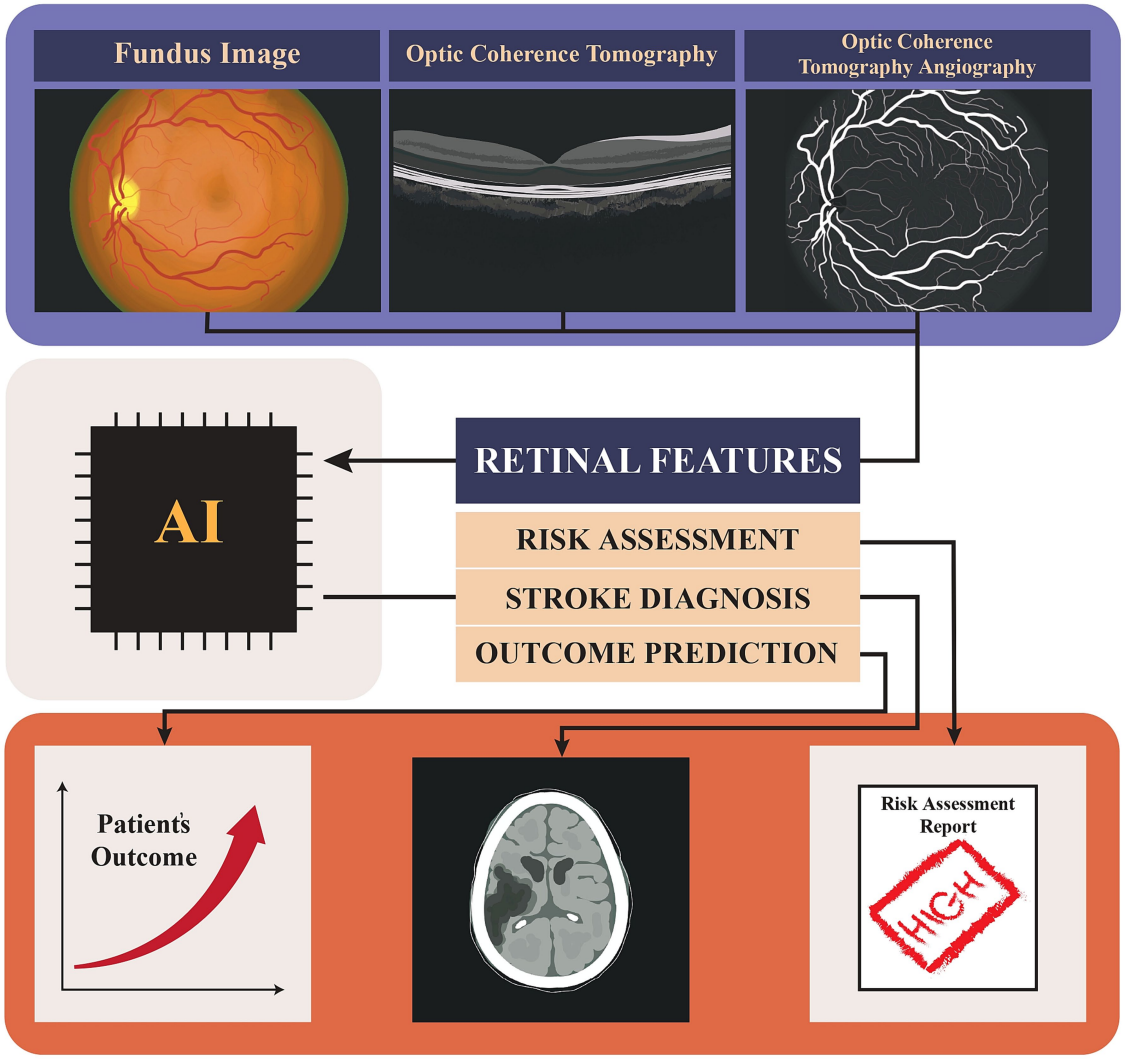
Graphical abstract showing the use of AI in analyzing retinal images (Fundus, Optical Coherence Tomography (OCT), and Optical Coherence Tomography Angiography (OCTA) for stroke risk assessment, diagnosis, and outcome prediction, ultimately contributing to patient outcome evaluation and risk assessment reporting.

This narrative review aims to explore the evolving role of AI in stroke patient care, with a particular focus on retinal imaging. We will highlight how AI can be integrated into clinical workflows to improve patient outcomes and propose strategies for addressing the challenges associated with this novel approach. To better understand the role of AI and retinal imaging in stroke, we will first summarize the retinal imaging techniques and AI models that were used. Then we will review the reported retinal changes related to stroke or stroke risk factors. Finally, we will overview the studies that used AI in stroke risk assessment and prognosis. Through this review, we seek to underscore the importance of continued research and innovation in the intersection of AI, retinal imaging, and stroke care, ultimately contributing to better health outcomes for patients at risk of stroke (Figure 1).

## 2 Methods

A comprehensive search was done on PubMed, Scopus, and Embase databases to find the relevant studies until July 2024. The used search terms were “artificial intelligence,” “deep learning,” “machine learning,” “stroke,” “retinal imaging,” “optic coherent tomography,” “retinal fundoscopy,” “ischemic stroke,” “hemorrhagic stroke,” “diagnosis,” and “prognosis.” In selecting articles for inclusion in our study, we focused on peer-reviewed original research published in English that clearly defined research objectives, employed rigorous methodologies, and investigated the use of different AI methods to analyze retinal images and use that data for risk assessment or diagnosis of stroke. We excluded non-peer-reviewed articles, those of low quality, studies with unclear research goals, duplicates, editorial articles, and conference abstracts.

## 3 The retina and retinal imaging

Different diseases affecting the retina can impose varying degrees of pattern distortions and dysfunctions in the retinal vasculature, changes in the thickness of the nerve fiber layer or ganglion cell layer, or cause pathologies in the optic disc, optic nerve head, or macula. Pathologic conditions will affect the thickness of different layers or the vasculature of the retina. Some of these conditions increase the risk of stroke and identifying them can help assess the patients’ risk for future stroke. DR, hypertensive retinopathy, central venous thrombosis (CVT), hyperhomocysteinemia, and systemic vasculitis are some of these conditions (Henderson et al., 2011; Gascon et al., 2018; Kunle-Hassan et al., 2018; Johannesen et al., 2019).

**Figure 2:**
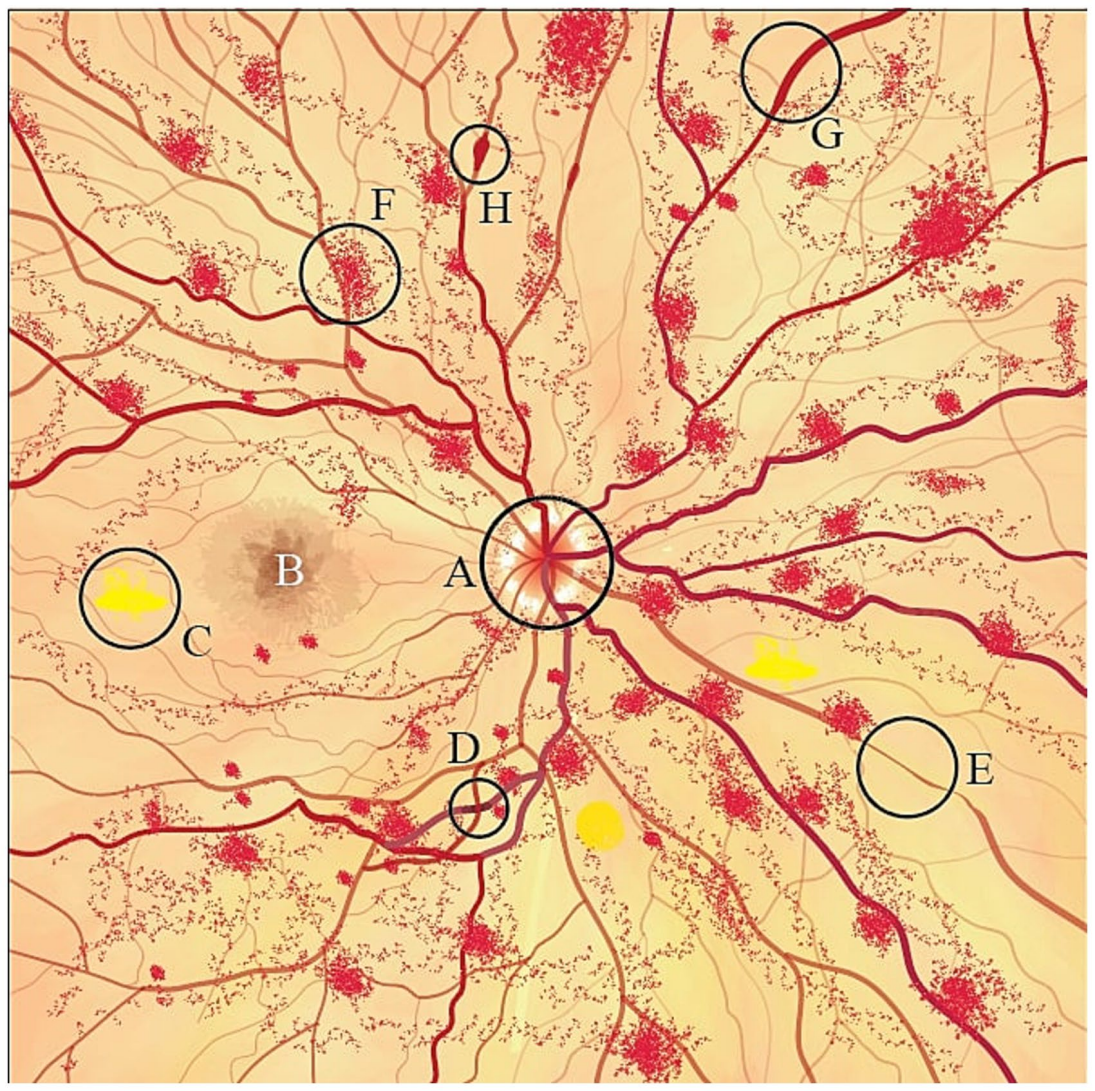
Schematic fondus illustration of retinal changes following a stroke. A, papilledema; B, macula; C, cotton wool spots; D, arteriovenous nicking; E, arteriolar narrowing; F, retinal hemorrhage; G, venous congestion; H, microaneurysms.

DR, as an adverse effect of DM affecting the retinal vessels, results in retinal changes, including microaneurysm and vessel wall leakage leading to focal and diffuse macular edema, respectively, (Bazargani et al., 2024; Sakini et al., 2024). Additionally, neovascularization of the optic disk and retina, retinal vessel hemorrhage in moderate to severe non-proliferative DR (NPDR), and thickening of RNFL are the other changes observed in DR (Chen et al., 2016; Johannesen et al., 2019; von Hanno et al., 2022). Hypertensive retinopathy, which can be used as a predictor of microvascular damage to the brain, presents as decreased capillary density, retinal arterial constriction, and hyperplasia of all arterial layers in early stages followed by an arterial dilation and plasma leak into the retina. This event is known as the exudative phase and could lead to vessel rupture and retinal bleeding. Arteriovenous nicking and cotton wool spots are other frequently seen abnormalities in the ophthalmoscopic examination of hypertensive retinopathy patients (Henderson et al., 2011; Johannesen et al., 2019; Tan et al., 2021). The retinal changes secondary to CVT are mostly RNFL and macular thinning. These changes result from increased intracranial pressure due to impaired venous return and can occur in CVT patients without papilledema on ophtalmoscopy (Koban et al., 2019). Additionally, case reports have described retinal hemorrhage as a secondary effect of CVT (Hauser et al., 1996; Kunle-Hassan et al., 2018). Systemic vasculitis syndromes can affect the retina primarily due to the underlying inflammatory changes in retinal vasculature. These conditions, including systemic lupus erythematosus (SLE), are often characterized by reduced vessel density and an enlarged foveal avascular zone, typically associated with venous and arterial occlusions leading to ischemia, hemorrhage, and cotton wool spots observed during fundoscopy (Gascon et al., 2018; Ji et al., 2022). Hyperhomocysteinemia, a metabolic condition that damages the endothelium, has been linked to retinal changes, including retinal pigment epithelium (RPE) disruption and retinal artery and vein occlusions (Wright et al., 2008; Lee et al., 2018; Figure 2).

Color Fundus Photography (CFP) and Optical Coherence Tomography (OCT) are critical non-invasive imaging modalities used to assess retinal microvasculature, with significant potential in predicting cerebrovascular events such as stroke (Raja et al., 2020; Watanabe et al., 2022). CFP captures two-dimensional retinal images using visible light, but its utility is constrained by limitations in image resolution and depth perception (Critser et al., 2024). Conversely, OCT utilize low-coherence interferometry to produce high-resolution, cross-sectional images of the retina and choroid, enabling precise quantitative analyses of retinal layers and three-dimensional structural evaluations (Aumann et al., 2019; Watanabe et al., 2022). OCT’s relevance in neurology is profound, with specific modalities such as RNFL-OCT, Ganglion Cell Complex OCT (GCC-OCT), macular OCT, and anterior segment OCT playing a pivotal role in detecting neurodegenerative changes, including optic nerve fiber degradation, ganglion cell loss, macular distortions, and anterior segment abnormalities such as papilledema (Kardon, 2011; Querques et al., 2019; Santorini et al., 2022). OCT angiography (OCTA) further extends these capabilities by utilizing motion contrast from blood flow to create detailed visualizations of retinal and choroidal vascular networks, offering a non-invasive means to study microvascular pathology at a capillary level (de Carlo et al., 2015; Rocholz et al., 2019). Despite these advancements, OCT and OCTA are limited by their narrower fields of view compared to CFP and involve higher operational costs (Niederleithner et al., 2022). On the other hand, Spectral-Domain OCT (SD-OCT) captures superior images more readily and uses a broadband multichromatic beam in order to detect complicated interference patterns to acquire high-quality dynamic images of the ocular structures (Hassenstein and Meyer, 2009). Swept-source OCT (SS-OCT), building on SD-OCT’s advantages, yields heightened sensitivity and decreased signal-to-noise ratio in deeper retinal layers (Baumann et al., 2012).

The integration of AI into retinal imaging represents a paradigm shift in the field, with advanced AI-driven tools like quantitative analysis of retinal vessel topology and size (QUARTZ) (Rudnicka et al., 2022) and Automatic retinal image analysis (ARIA) (Qu et al., 2022), as well as deep learning (DL) algorithms such as U-net (Ronneberger et al., 2015), IterNet (Li et al., 2020), and OCTA-Net (Ma et al., 2021), significantly enhancing the segmentation of vascular structures and extracting critical parameters, including vessel diameter, density, tortuosity, and fractal dimension.

## 4 Frequently used AI models in the stroke and retinal imaging confluence

### 4.1 Machine learning models

Nowadays, AI is increasingly used for tasks that involve handling large datasets, automating repetitive functions, and ensuring consistency in categorization, classification, and prediction (Duan et al., 2019; Roh et al., 2021). Machine learning (ML) and deep learning (DL), key subfields of AI, are essential for enhancing medical image analysis (Burlina et al., 2019). In contrast to traditional AI methodologies, which depend on static, predefined algorithms, ML operates autonomously, identifying patterns within data and continuously refining its models without explicit programming (Gao et al., 2024). In the context of retinal and neuroimaging, ML can be used to delineate regions of interest within the images or classify them into specific categories, such as non-ischemic, IS, or HS images, aiding diagnosis and treatment planning (Mariano et al., 2022; Qu et al., 2022). Supervised ML involves training a model to map input features to corresponding output labels or values. Support vector machine (SVM), naive Bayes (NB), random forest (RF), k-nearest neighbor (KNN), and decision tree (DT) are the commonly featured ML structures in stroke and retinal imaging research topics.

KNN has been widely used for classifying types of strokes from neuroimaging data (Dourado et al., 2019), vessel segmentation (R and Balasubramanian, 2018; Rehman et al., 2022), detection of exudates (Ghalwash et al., 2017), and microaneurysms (Walter et al., 2007; Schmidt-Erfurth et al., 2018) in retinal images. Based on Bayes’ theorem, the NB Classifier calculates the probability of a hypothesis (or class) given the evidence (or features). Despite its simplicity, NB is commonly employed when computational efficiency and simplicity are prioritized and the assumption of feature independence is not too far from reality (Vikramkumar et al., 2014; Raschka, 2017). It has been used for segmenting ischemic lesions (Griffis et al., 2016), detecting cerebral microbleeds (Ateeq et al., 2023), post-stroke cognitive impairment (Ji et al., 2023), and predicting stroke reoccurrence (Wang et al., 2023; Gao et al., 2024).

SVM determines the optimal hyperplane decision boundary, effectively delineating binary classes, with the data points closest to this boundary termed support vectors. SVM approaches have been applied extensively in medical imaging for segmenting targets in CFP and OCT images such as vessels (Zawadzki et al., 2007; Ghalwash et al., 2017; Ghosh and Ghosh, 2021), microaneurysms (Veiga et al., 2018; Derwin et al., 2022), hard exudates (Jaya et al., 2015), drusen (Khalid et al., 2018), fovea (Liu et al., 2012), and neovascularization (Yu et al., 2016; Schmidt-Erfurth et al., 2018). In stroke management, SVM has been utilized to locate large vessel occlusions in neuroimaging data and predict stroke outcomes with or without endovascular treatment (van Os et al., 2018; Nishi et al., 2019; Fang et al., 2020; Rava et al., 2020).

DTs organize data into hierarchical tree-like structures suitable for decision-making in stroke management in a clinical setting. However, it has limitations such as overfitting, sensitivity to minor fluctuations in the data, and bias toward features with more levels (Quinlan, 1986; Song and Lu, 2015). To overcome the challenges of individual DTs and enhance predictive accuracy, ensemble methods combine multiple relatively weak DTs to form a robust ensemble model. RFs train each tree independently and in parallel, utilizing a random subset of the data through bootstrapping. Subsequently, predictions from individual trees are aggregated to produce the final prediction (Breiman, 2001; Schonlau and Zou, 2020). RFs have been used for the segmentation of retinal layers (Lang et al., 2013), drusen (van Grinsven et al., 2013), pseudodrusen (van Grinsven et al., 2015), exudates (Liu et al., 2011), and geographic atrophy (Feeny et al., 2015; Schmidt-Erfurth et al., 2018). In the context of stroke, RF has been applied to segment and measure cerebrospinal fluid volume (Dhar et al., 2018) and ischemic lesions (Mitra et al., 2014), estimate penumbra volume (McKinley et al., 2017), predict long-term stroke outcomes (van Os et al., 2018; Heo et al., 2019; Fernandez-Lozano et al., 2021), and design personalized upper extremity rehabilitation (Camardella et al., 2022).

In contrast to RF, Gradient Boosting and AdaBoost adopt a sequential approach, where DTs are constructed sequentially to rectify errors made by preceding trees. Gradient Boosting focuses on minimizing a loss function by adjusting predictions of each successive tree to correct residuals left by previous trees (Natekin and Knoll, 2013). They have also been utilized for the detection of intravascular filling defects on fluorescein angiogram images (Zhao et al., 2015), quantification of choroidal neovascularization (Tsai et al., 2011), and identification of DR (Jiang et al., 2019).

Multilayer perceptron (MLP) serves as foundational architectures for more advanced neural networks such as convolutional neural networks (CNNs), and recurrent neural networks (RNNs). MLP introduces nonlinearity into the network via the use of non-linear activation functions such as sigmoid, hyperbolic tangent (tanh), and rectified linear units (ReLU) (Murtagh, 1991; Popescu et al., 2009). MLPs have been used for the assessment of retinal vascular branching (Atagun et al., 2022), segmentation of hard exudates (García et al., 2009), as well as predicting hemorrhagic transformation in MRI images (Elsaid et al., 2022) and identifying stroke mimics in prehospital triage (Zhang Z. et al., 2022).

### 4.2 Deep learning models

DL draws inspiration from complex neural networks found in the human brain, consisting of many interconnected layers of nodes (neurons) in its well-designed hidden layers, with each node receiving weighted input from nodes in the previous layer, performing predefined activation function on the data and sending output to neurons in the next layer (Han et al., 2018). It excels at acquiring hierarchical data representations through multiple layers of abstraction, drawing non-linear mapping from features to outcomes. Among the different types of DL algorithms, including autoencoders, generative adversarial networks (GANs), CNN, and RNN, CNNs are the most frequently used DL infrastructures.

CNNs are tailored to excel in tasks such as feature extraction, image classification, object detection, and image segmentation (Xu and Zhang, 2022; Derry et al., 2023). The CNN algorithm, as its name implies, utilizes a process called convolution. Feature extraction begins by applying convolution (filter multiplication) to the input data, yielding feature maps. Subsequently, the resulting feature maps undergo a non-linear activation function, typically ReLU, followed by pooling to reduce spatial dimensions and enhance computational efficiency, thereby preserving essential features while mitigating overfitting. The pooling stage also increases the field of view of convolutional kernels and encourages learning more abstract and global features. The flattened data derived from the feature extraction stage is then channeled into a fully connected neural network for classification. Herein, the network learns to differentiate between different classes based on the extracted features. An activation function and a corresponding loss function are applied in the final classification stage (Xu and Zhang, 2022; Derry et al., 2023). CNN architectures such as VGG (Simonyan and Zisserman, 2015), Inception (Szegedy et al., 2016), Xception (Extreme Inception) (Chollet, 2017), and U-Net (Ronneberger et al., 2015) are notable in retinal and stroke imaging.

While VGG is acknowledged for its architectural straightforwardness and performance using compact 3×3 filter sizes supplemented by max-pooling layers for spatial down sampling, its deep configuration results in notable computational and memory requirements during training and evaluation. These demands could impede practical deployment in real-world applications (Simonyan and Zisserman, 2015; Szegedy et al., 2016). Despite this, it has been adopted for various tasks, such as the detection of DR (Ting et al., 2017), differentiation of active corneal ulcers from healed scars (Tiwari et al., 2022), stroke classification from MRI and CT images (Szegedy et al., 2016; Chen Y.-T. et al., 2022; Abbaoui et al., 2024), and prediction of functional outcomes of stroke (Lai et al., 2022). In contrast to VGG, Inception, by applying an additional 1×1 filter and substituting fully connected layers with a global average pooling layer, balances computational efficiency and model performance, making Inception networks well-suited for scenarios with restricted computational resources and extensive datasets. Inception-v3 further refines this approach, by replacing larger convolutions with combinations of smaller ones, reducing parameters and computational complexity while maintaining performance standards (He et al., 2016; Szegedy et al., 2016). Additionally, Xception further decomposes the convolution operation into smaller, more manageable components, fostering parameter sharing within localized regions of the input rather than across the entire dataset (Chollet, 2017).

ResNet addresses the vanishing gradient problem by introducing skip or residual connections, empowering the training of very deep (especially over 50 layers) networks without suffering from performance degradation typically associated with increased depth. However, this advancement comes at the expense of heightened computational requirements and increased susceptibility to overfitting specially when the model is trained on smaller datasets (Ebrahimi and Abadi, 2021). Nonetheless, the availability of pre-trained ResNet models simplifies transfer learning (TL) for various applications (He et al., 2016).

The U-Net architecture is distinguished by its U-shaped structure arising from the symmetrical arrangement of its contraction (encoding) and expansion (decoding) paths. Additional connections between encoder and decoder layers allow images to be processed at different levels of abstraction. It has garnered extensive application in medical image segmentation and detection tasks, even when confronted with limited training data (Ronneberger et al., 2015; Hemelings et al., 2019). ReLayNet, a variant inspired by U-Net, has achieved accurate segmentation of seven retinal layers and associated fluid in pathological OCT scans (Roy et al., 2017; Schmidt-Erfurth et al., 2018).

IterNet (Li et al., 2020) and OCTA-Net (Ma et al., 2021) are specialized architectures tailored for retinal vessel segmentation in CFP and OCTA images. IterNet incorporates multiple iterations of a mini-UNet architecture, resulting in a network that is 4 times deeper. Conversely, OCTA-Net utilizes ResNet as its backbone architecture. Both IterNet and OCTA-Net have demonstrated slight improvements in performance compared to their foundational CNN algorithms, such as U-Net and Residual U-Net, in retina vessel segmentation tasks. Specifically, IterNet achieved an area under the curve (AUC) of 0.981, outperforming U-Net (AUC = 0.975) and Residual U-Net (AUC = 0.977). Similarly, OCTA-net achieved an AUC of 0.937, surpassing U-Net (AUC = 0.903) and Residual U-Net (AUC = 0.910) (Li et al., 2020; Ma et al., 2021).

Databases like the UK Biobank, Retinal OCTA Segmentation dataset (ROSE), and Anatomical Tracings of Lesions After Stroke (ATLAS) provide essential resources for training DL models; however, limited sample sizes present a significant challenge in their development (Liew et al., 2018; Ashayeri et al., 2024a). TL addresses this issue by utilizing a pre-trained model for one task, which is then adapted for a second task. TL has been shown to enhance model performance while reducing the need to increase the sample size (Chen J. et al., 2022; Ashayeri et al., 2024b). The GAN is a method that uses two CNN models: one generator and one discriminator. The generator creates images that mimic the original images, and the discriminator tries to identify the original images from fake images. The trained generator in GAN can be useful in tasks such as increasing image quality. However, the memory requirements of this method are high (Wang et al., 2021).

## 5 Retinal biomarkers in stroke

Retinal changes associated with stroke can be divided into two categories: those caused by stroke risk factors and those that indicate stroke risk independently of known risk factors. The latter category includes changes observed in stroke patients without any known risk factors and can serve as a tool for future stroke risk assessment (Cheung et al., 2013; Boehme et al., 2017; Reza et al., 2024). Identifying these changes can help researchers develop models to assess them in suspected patients. Recognizing these biomarkers with AI methods can lead to faster and more accurate stroke management.

Notably, a decrease in the central retinal artery (CRA) diameter and an increase in the central retinal vein (CRV) diameter are associated with both lacunar stroke and intracranial hemorrhage (Cheung et al., 2017). Specifically, a systematic review and meta-analysis involving 12,919 subjects demonstrated that decreased CRA diameter and increased CRV diameter resulted in hazard ratios (HR) of 1.18 (1.04–1.34) and 1.20 (1.10–1.31), respectively, for stroke (Girach et al., 2024). AI models, such as SVM-based models, can accurately detect the vessel diameter based on the retinal images and use the information to differentiate the high-risk vs. low-risk group (Barkana et al., 2017). Increased vessel tortuosity, arteriovenous nicking, and enhanced arteriolar light reflex have also been reported in stroke patients (Cheung et al., 2017). A study showed that straighter retinal arterioles was co-related with stroke risk (HR = 0.38); however, this linkage lost its significance when arteriolar tortuosity was considered as a continuous variable (Cheung et al., 2013). Decreased retinal fractals are also reported to be associated with stroke (odds ratio (OR): 1.85–2.28) (Wu et al., 2017). Additionally, according to Ong et al., the arteriolar network has been suggested to have a stronger association with stroke in comparison to the venular network (OR: 2.28 vs. 1.8) (Ong et al., 2013a). Even after controlling for conventional cardiovascular risk factors, Liew et al. report an HR of 1.26 for stroke as retinal fractal decreases (Liew et al., 2021). Models that excel at image segmentation and feature extraction, such as CNN, may have an advantage in detecting venous form arterioles and degree of vessel tortuosity.

A study in patients with type 2 DM reveals that a higher fractal dimension is associated with a lower risk of stroke (Sandoval-Garcia et al., 2021). RNFL defects are indicators of acute or previous stroke in IS patients (Wang et al., 2014; Liang et al., 2022). Retinal vessel occlusion, such as central retinal artery occlusion (CRAO), central retinal venous occlusion (CRVO), and branch retinal vein occlusion (BRVO), are independent predictors of stroke due to their embolic origins (Dumitrascu and Koronyo-Hamaoui, 2020; Rim et al., 2020). Microvascular changes like arteriovenous nicking, arterial narrowing, and venous dilation are especially associated with lacunar infarction and higher stroke risk by two to threefold (Dumitrascu and Koronyo-Hamaoui, 2020). In contrast to these findings, another study reports severe arteriovenous nicking is associated with lobar ICH compared to lacunar infarcts (Baker et al., 2010).

Several diseases are proposed to alter the risk of cerebrovascular accident (CVA), and concomitantly affect the retina. Some of these risk factors are closely associated with metabolic syndrome, like high body mass index (BMI), DM, smoking, HTN, and hyperhomocysteinemia. Smoking and high BMI are associated with RNFL thinning (Yang et al., 2019; Salehi et al., 2022), and are also risk factors for DM and HTN. A study, which utilized dual-energy X-ray absorptiometry to measure body fat also reported no thinning in the RNFL layer. Instead, ganglion cell-inner plexus layer (GCIPL) thinning was notable. It appears that BMI is associated with RNFL and GCIPL thickness, but body fat percentage is only associated with the GCIPL layer (von Hanno et al., 2022). For further evaluation of the smoking effect, a study used OCTA to detect blood flow changes after smoking a single cigarette and reported a significant reduction, especially in the first 5 min of smoking. However, the relationship between this reduction and cerebrovascular accidents (CVA) due to long-term cigarette use remains unclear, necessitating further research (Ayhan et al., 2017).

**Figure 3:**
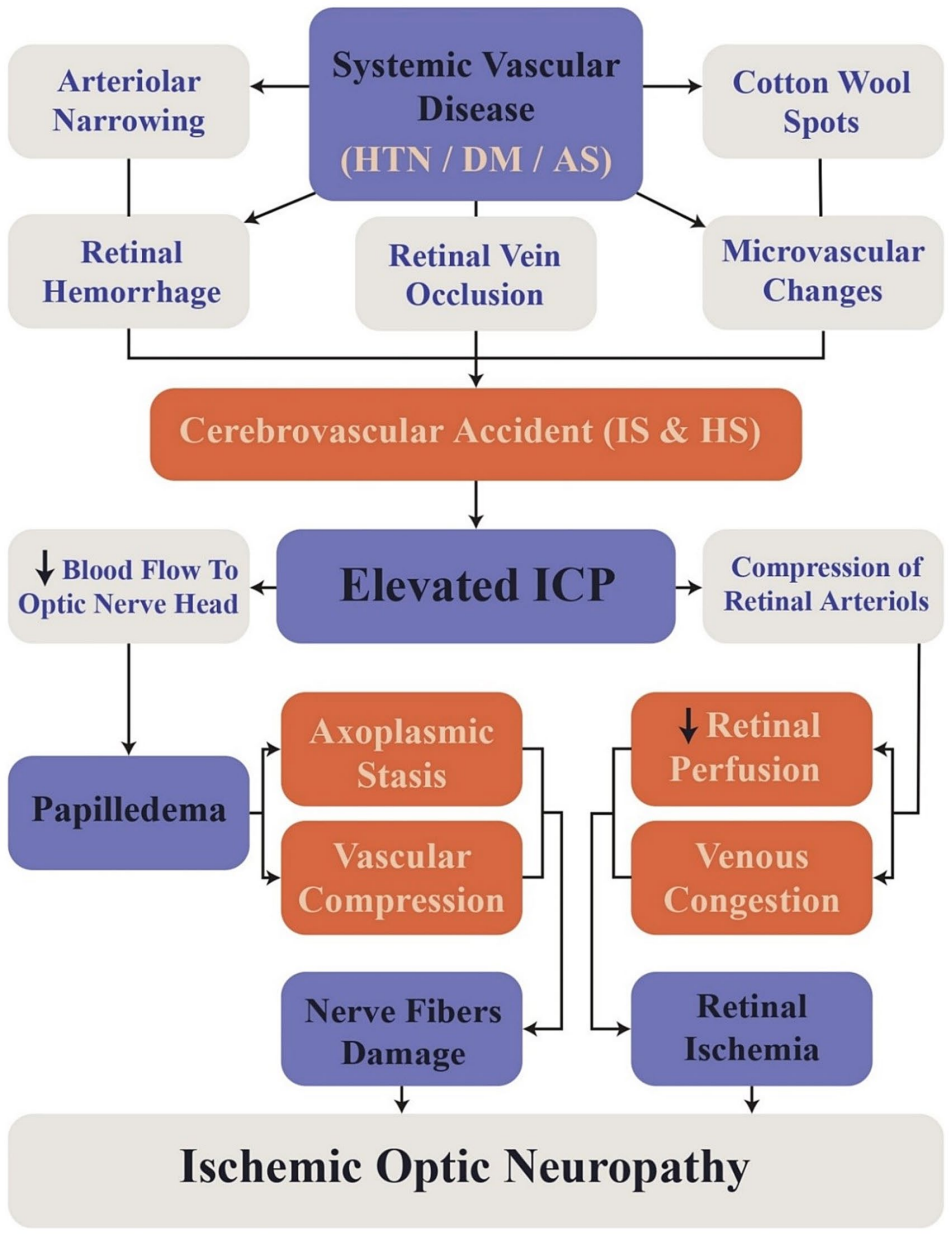
Flowchart illustrating the sequence of events linking retinal changes to cerebrovascular accidents. HTN, Hypertension; DM, Diabetes Mellitus; AS, Atherosclerosis; ICP, Intracranial Pressure; IS, Ischemic Stroke; HS, Hemorrhagic Stroke.

DM and HTN cause retinopathy due to microvascular damage to retinal vessels (Henderson et al., 2011; Barot et al., 2013), increasing stroke risk (either hemorrhagic or ischemic) as the retinopathy stage progresses (Ong et al., 2013b; Hu et al., 2021; Wang et al., 2022). Two systematic reviews investigate the role of DR in stroke in type II DM revealed that the severity of DR is positively correlated with an increased likelihood of ischemic stroke, particularly microvascular IS. In the first review, HR for moderate NPDR was 2.08 times than that of mild NPDR. Similarly, the second review reported an HR of 2.01 for mild NPDR and 2.27 for severe NPDR (Hu et al., 2021; Wang et al., 2022). A study conducted over 2 years on HS patients due to primary HTN reports that over half of HS patients have high-stage hypertensive retinopathy compared to primary HTN patients without any previous HS. The higher stages of hypertensive retinopathy are associated with lower glasgow coma scores (GCS) at admission, which also worsens the prognosis (Thiagarajah et al., 2021). The study also reported that higher stages of retinopathy were linked to higher rates of hemorrhages with clots larger than 30 mL, which may reflect the extent of hemorrhage and correlate with GCS scores (Thiagarajah et al., 2021; Figure 3). Although each model hypothetically may have an advantage in extracting specific data, in real-life situations there are multiple factors determining the performance of architectures. Some of these variables are the use of pre-trained models, the number of training and testing sets, methods used to avoid overfitting or underfitting, and the computational power each model requires (Daich Varela et al., 2023).

## 6 AI-aided retinal biomarker detection in stroke risk-appraisal, diagnosis, and prognosis

It has been observed that concrete associations could be found between distinct retinal features and stroke identification or risk assessment. With further probing of the unidentified aspects of the field, the stroke risk estimation and diagnosis using retinal image analysis will rely on the development of intricate models encompassing a multitude of retinal features and data obtained from stroke patient history, physical examination, laboratory data, or various neuroimaging modalities. The AI models can use multiple inputs and extract features from retinal images which is useful in stroke risk assessment and diagnosis.

### 6.1 Risk assessment

Recent studies have utilized AI-based approaches to enhance the predictive abilities of retinal imaging for stroke incidence, with varying results. Stroke risk assessment could be achieved using conventional globally renowned scoring systems, including CHA_2_DS_2_-VASc or FRS, combined with novel AI tools or innovative risk evaluation algorithms using ML and DL, including retinal age gap and retinal vascular fractal dimension’s association with stroke risk.

A recent effort to combine DL algorithms with established stroke risk-stratifying assessment frameworks was conducted by Germanese et al. (2024), resulted in a reliable classification of 491 patients into low or intermediate-high stroke risk groups (AUC = 0.71–0.96) Additionally, the model predicted the CHA_2_DS_2_-VASc risk score with a maximum accuracy of 68%. The analysis was based on retinal vascular distortions observed in SS OCT-A images, using the EfficientNetV2-B3 tool. Similarly, Rudnicka et al. (2022) compared the predictive performance of the FRS with and without the addition of AI-assisted retinal vasculometry. They developed models using supervised ML and DL, trained on the UK Biobank cohort (*n* = 88,052), and validated externally in the EPIC Norfolk cohort (*n* = 7,411). QUARTZ system (Rudnicka et al., 2022) automatically extracted retinal vessel width, tortuosity, and total vessel area, with a SVM assessing image quality and DL distinguishing arterioles from venules. Despite this advanced integration, the addition of retinal vasculometry did not significantly enhance model performance (UK Biobank: C-statistic of 0.74 for FRS-only vs. 0.73 for men and 0.75 for women in FRS-RV; EPIC Norfolk: C-statistic of 0.68 for men and 0.73 for women in FRS-only vs. 0.691 for men and 0.714 for women in FRS-RV). While the study benefited from a large sample size and external validation, its generalizability may be limited due to the predominantly white cohort used for model development.

A novel predictor of stroke risk called “retinal age gap” was introduced by Zhu et al. (2022) to explore an alternative AI application. Zhu’s study focused on a cohort from the UK Biobank (*n* = 11,052) to predict individual retinal age from fundus photography and OCT, by using the Xception DL model (Chollet, 2017). They assessed risk of stroke among 35,304 stroke-free participants by measuring the retinal age gap, defined as the difference between predicted retinal age and chronological age. Their findings showed that each one-year increase in the retinal age gap corresponded to a 4% increase in stroke risk (HR = 1.04, 95% CI: 1.00–1.08, *p* = 0.029), with those in the highest quintile of the retinal age gap exhibiting a significantly higher stroke risk (HR = 2.37, 95% CI: 1.37–4.10, *p* = 0.002). The study’s strengths include its large sample size, extended follow-up period, and comprehensive adjustment for confounding variables. However, its limitations, such as the lack of ethnic diversity, absence of external validation, and the relatively youthful cohort, suggest the need for further research to confirm these findings across more diverse populations. The predictive ability of retinal age alone was found to be comparable to that of the established FRS (AUC = 0.676 vs. AUC = 0.661, *p* = 0.511). Notably, Zhu et al. (2022) achieved lower accuracy for FRS while using the same dataset compared to Rudnicka et al. (2022), which may be due to the smaller dataset size they used (63,839 vs. 35,304). This suggests that the “retinal age gap” model’s accuracy could be enhanced by training on a larger, more diverse dataset.

Taking the investigation further into innovative predictive modeling, Li et al. (2023) explored the potential of multi-spectral fundoscopic imaging combined with DL to predict IS risk within 1 year in patients with AF. By employing models like Inception V3, ResNet50, and SE50, Li et al. built upon the foundational AI applications demonstrated by Rudnicka et al. (2022) and Zhu et al. (2022), but focused specifically on the predictive capabilities in a clinical context. They trained their models on 150 participants with AF and no history of IS, along with 100 participants with IS of unknown cause or recent AF diagnosis. The study demonstrated robust predictive abilities across all models, with the lowest AUC and accuracy values at 0.863 and 0.785, respectively. The multi-spectral Inception V3 model achieved the highest performance, with an AUC of 0.954 and an accuracy of 0.918. The models performed consistently better with 605 nm spectral images and multi-spectral data compared to single-spectral inputs. Despite these strengths, the study’s limitations included age-related confounding due to the older AF group, lack of validation sets, and a relatively small training sample size.

Additionally, Qu et al. (2022) applied a RF model to retinal images to assess stroke risk and differentiate IS from HS. The study involved 231 stroke patients (145 IS, 86 HS) and 480 controls. Retinal features were extracted using ResNet50 CNN and the ARIA algorithm (Qu et al., 2022), focusing on texture and fractal dimensions associated with stroke subtypes. Logistic regression analysis showed that retinal characteristics alone provided superior predictive performance (AUC = 0.98 for both IS and HS) compared to clinical characteristics alone (AUC = 0.88 for IS, 0.91 for HS). The RF classifier demonstrated a sensitivity of 91.0%, specificity of 94.8%, AUC of 0.929 for ischemic stroke, and a sensitivity of 93.0%, specificity of 97.1%, and AUC of 0.951 for HS. While the study successfully differentiated between stroke subtypes, its limitations include a small training dataset, lack of external validation, and a focus on intracerebral hemorrhage, which may limit generalizability to other HS types.

### 6.2 Diagnosis

In a comparable effort to improve diagnostic capabilities, Raveendran Susha et al. (2020) utilized AI to analyze retinal vascular features for stroke prediction. By using the Vessel Assessment and Measurement Platform for Images of the Retina (VAMPIRE) annotation tool (Perez-Rovira et al., 2021) to assess a dataset of 130 retinal images, they identified key features such as fractal dimension and branching coefficients, achieving high accuracy with the NB classifier (AUC of 0.976 and accuracy of 0.965). However, the study’s small sample size and single-source data limited its applicability.

Further expanding on diagnostic improvement topic, Lim et al. (2019) applied the VGG19 DL architecture (Simonyan and Zisserman, 2015) to predict IS using a diverse dataset of retinal images from multiple sources. They utilized both “templated” images (with noise reduction and border standardization) and “vessel” images (segmented using U-Net; Ronneberger et al., 2015) to analyze 4,528 stroke-positive images from the Multi-Centre Retinal Stroke (MCRS) study and 6,622 stroke-negative images from five other datasets. The study deployed three experimental setups: E-All (all six datasets), E-Split1 (Singapore datasets for negative images), and E-Split2 (Melbourne and Singapore datasets for negative images). While the E-All-Templated model initially performed best (AUC = 0.987), it exhibited a significant drop in accuracy when tested on geographically diverse datasets, indicating potential overfitting to environment-specific features. This concern echoed the need for better generalization, as highlighted by Rudnicka et al. (2022) and Zhu et al. (2022). To mitigate this issue, the study employed vascular tree segmentation to enhance generalizability, though this approach resulted in slightly lower AUCs (E-All-Vessel AUC = 0.855). Despite the improved generalizability of vessel images, the study faced limitations, including a small number of stroke-positive images, reliance on a single source for these images, and a lack of diversity in stroke-negative images, all of which contributed to overfitting and limited the model’s broader applicability.

Coronado et al. (2021) addressed this issue by employing a different approach, using vasculature embeddings combined with DT gradient boosting (LGBM). Vasculature embeddings, derived from a U-shaped neural network and fine-tuned with LGBM, were evaluated using fundus images from 2,060 stroke-free individuals and 412 stroke patients from the UK Biobank. The vasculature embeddings achieved an AUC of 0.626, outperforming VGG19 (AUC = 0.548) and Inception-v3 (AUC = 0.499), while also being more computationally efficient. In an age-restricted cohort, VGG19 showed improved performance (AUC = 0.714), but LGBM achieved similar accuracy (AUC = 0.674) with fewer parameters. Inception-v3 underperformed across datasets, likely due to its higher-dimensional feature vector, which may have led to overfitting to real-world image features rather than stroke-specific patterns.

Pachade et al. (2022) used ResNet-50 in a self-supervised contrastive learning approach to extract features from OCT-500 and ROSE datasets, including CFP and OCTA images. Analyzing 112 retina images from 16 stroke patients and 73 controls, they refined the cohort to 15 IS patients and 21 age-matched controls. Vessel segmentation was performed using IterNet and OCTA-Net, followed by extraction of fractal dimension and macular vessel density features. The highest AUCs were achieved for microvasculature density by using KNN (*k* = 9) with leave-one-subject-out (LOSO) validation strategy (0.87 in the full cohort, 0.88 in the age-controlled cohort). Fractal dimension was significant only in fundus images (AUC = 0.57 full, 0.72 age-controlled) and multimodal images (AUC = 0.70 full). The self-supervised model, which employed momentum contrast (MoCo) with Mean Square Error (MSE) loss, performed better than MoCo with Barlow Twins (BT) loss in feature extraction, especially in the full dataset. Moreover, KNN achieved the best overall AUCs of 0.81 (full cohort) and 0.66 (age-controlled), followed by NB (0.76), RF (0.74), and others. The study’s strengths include the use of multimodal imaging near stroke occurrence, but it was limited by a small sample size, inability to distinguish stroke types, and low-quality images.

Zhou et al. (2023) developed the “RETFound” Self-Supervised Learning (SSL) model using CFP and OCT images to detect ocular conditions like DR and glaucoma, as well as systemic diseases including IS and myocardial infarction. The model combined generative SSL with contrastive methods such as SimCLR (Chen et al., 2020), SwAV (Caron et al., 2020), DINO (Caron et al., 2021), and MoCo-v3 (Chen et al., 2021). They compared three pre-trained models: SL-ImageNet, SSL-ImageNet, and SSL-Retinal. The RETFound-MAE model was trained sequentially on natural images from ImageNet-1 k and then on 904,170 CFP and 736,442 OCT retinal images, primarily from the Moorfields Diabetic Retinopathy Dataset, with image classification performed by MLP. Performance was validated internally using the AlzEye dataset (353,157 subjects) and externally with the UK Biobank (82,885 subjects). RETFound-MAE performed well in detecting ocular diseases but showed limited accuracy in predicting systemic diseases, particularly stroke, during external validation. The AUROC for AlzEye was 0.75 for both CFP and OCT images, but it dropped to 0.59 for CFP and 0.56 for OCT in the UK Biobank, suggesting overfitting and shortcut learning. Despite this, RETFound-MAE outperformed other strategies in AlzEye for stroke prediction, while RETFound-DINO performed better in the UK Biobank, though neither matched the internal dataset’s performance. RETFound-MAE also exceeded the performance of other pre-trained models in both datasets. Strengths of the study include diverse cohorts, a large training set, external validation, and the inclusion of both CFP and OCT images. However, limitations involve the model’s development primarily on a diabetic cohort, lack of multimodal imaging for model development, and the geographical concentration of the training dataset within the UK.

### 6.3 Outcome prediction

Leptomeningeal collateral circulation, which connects cerebral artery branches, is critical in IS outcomes, with poor collateral status leading to larger infarctions and higher mortality (Maguida and Shuaib, 2023). Khan et al. (2022) utilized SVM on retinal vessel parameters to predict collateral status in 35 stroke patients with middle cerebral artery occlusion and 21 healthy controls. Collateral status was graded by computed tomography angiography on a scale of 0 (poor) to 3 (good) following the criteria outlined by Tan et al. (2009). Using principal component analysis for dimensionality reduction, the SVM model achieved 74.3% accuracy, 74.3% sensitivity, and 70.7% specificity. The study’s strengths include double-blinded, semi-automated retinal vessel analysis, though it was limited by a notably small sample size, lack of external validation and inclusion of only moderate stroke patients. Table 1 summarizes the various AI model performances across different studies and datasets.

**Table 1:** Summary of the performance of AI models for assessment of stroke via retinal imaging.

References	Year	Total number of samples (Stroke-negative and Stroke-positive or Stroke-negative/IS/HS)	Observation method	AI algorithm	Database	Results
Risk assessment						
Qu et al. (2022)	2022	711 (480/145/86)	Retinal photography	ARIA and ResNet50 (feature extraction), RF (stroke subtype classification)	Shenzhen Traditional Chinese Medicine Hospital	1. IS risk estimation: Sensitivity: 91.0% Specificity: 94.8% AUC: 0.929 (95% CI: 0.900 to 0.958) 2. HS risk estimation: Sensitivity: 93.0% Specificity: 97.1% AUC: 0.951 (95% CI: 0.918 to 0.983)
Zhu et al. (2022)	2022	35,304 (35,022/282)	Fundus photography—OCT	Xception (prediction of retinal age)	UK Biobank	1. Retinal age gap (Xception-predicted age minus chronological age): AUC = 0.676 2. Risk-factor model: AUC = 0.661
Rudnicka et al. (2022)	2022	Training: 63,839 (63,393/446) External Validation: 5,708 (5,497/211)	Fundus photography	QUARTZ (feature extraction), SVM (image quality score), CNN (arteriole/venule classification)	UK Biobank (training), EPIC-Norfolk (external validation)	1. AI-Enabled Retinal Vasculometr: C-statistic = 0.66–0.77 R^2^ statistics = 0.17–0.39 2. FRS: C-statistic = 0.67–0.77. R^2^ statistics = 0.2–0.43.
Li et al. (2023)	2023	250 (150/100/0)	Multi-spectral fundus photography	Inception V3, ResNet50 and SE50 (stroke classification)	Chinese Han population	1. Accuracy > 0.78 for IS risk prediction (secondary to AF). 2. Multi-spectral models outperformed single-spectral models (the highest AUC = 0.954).
Germanese et al. (2024)	2024	491 (225 low neurocardiovascular risk, 266 intermediate–high neurocardiovascular risk)	SS OCT-A	ML: DT, RF, SVM, logistic regression DL: EfficientNetV2-B3 (predict the CHA2DS2-VASc neurocardiovascular risk)	RASTA	ML Models (SS OCT-A + Clinical Data):1. SVM (AUC = 0.98, accuracy = 0.851) 2. logistic regression (AUC = 0.96) 3. RF (AUC = 0.91) 4. DT (AUC = 0.78)DL Model (SS OCT-A Only):1. EfficientNetV2-B3 (accuracy = 0.68) 2. RF variants (accuracy = 0.61 and 0.54)
Diagnosis						
Lim et al. (2019)	2019	11,150 (6,622/4528/0)	Fundus photography	VGG19 (stroke classification)	Stroke-positive: MRCS Stroke-negative: SCES, SiMES, SiNDI, SP2, DMPMelb	Templated Model Performance: 1. Overall Performance: AUC ≥ 0.966 for stroke prediction. 2. Feature Isolation: AUC = 0.754–0.855 with vessel images. 3. Dataset Ablation: AUC = 0.496–0.994 on unseen data.
Raveendran Susha et al. (2020)	2020	130 (80/50/0)	Fundus photography	SVM, MLP, RF, DT, NB (stroke classification)	Sree Gokulam Medical College and Research Foundation	NB classifier: 1. Accuracy = 0.9692 2. AUC = 0.968 SVM: Accuracy = 0.89 RF: Accuracy = 0.8977 DT: Accuracy = 0.873 MLP: Accuracy = 0.857
Coronado et al. (2021)	2021	Full: 2,472(2,060/412) age-restricted: 1,200 (1,001/199)	Fundus photography	LGBM, VGG19, Inception-v3 (stroke classification)	UK Biobank	1. The vasculature embedding-LightGBM model: AUROC = 0.626 2. The vasculature embedding-LightGBM model (age-restricted dataset): AUROC = 0.674 3. VGG19: AUROC = 0.548 4. VGG19 (age-restricted dataset): AUROC = 0.714 5. Inception-v3: AUROC = 0.499 6. Inception-v3 (age-restricted dataset): AUROC = 0.512
Pachade et al. (2022)	2022	89 (73/15/1) + 729 Unlabeled	Fundus Photography, OCT-A	Iternet, OCTA-Net (Vessel segmentation), PCA and KNN (feature engineering), ResNet50 (self-supervise learning), KNN, DT, RF, MLP, AdaBoost, Gaussian NB (stroke classification)	Memorial Hermann Texas Medical Center, OCT-500, ROSE	1. Feature Engineering: AUC = 0.87 –0.88, with fractal dimension features showing no significant impact. 2. Self-Supervised Learning: Momentum contrast approach achieved AUC of 0.81 (full cohort) and 0.66 (age-stroke-controlled cohort). 3. Supervised Classifiers (Self-Supervised Features): KNN: AUC = 0.81 RF: AUC = 0.78 DT: AUC = 0.75 MLP: AUC = 0.74 AdaBoost: AUC = 0.72 Gaussian NB: AUC = 0.68
Rudnicka et al. (2022)	2022	Training: 63,839 (63,393/446) External Validation: 5,708 (5,497/211)	Fundus photography	QUARTZ (feature extraction), SVM (image quality score), CNN (arteriole/venule classification)	UK Biobank (training), EPIC-Norfolk (external validation)	1. AI-Enabled Retinal Vasculometr: C-statistic = 0.66–0.77 R^2^ statistics = 0.17–0.39 2. FRS: C-statistic = 0.67–0.77. R^2^ statistics = 0.2–0.43.
Zhu et al. (2022)	2022	35,304 (35,022/282)	Fundus photography - OCT	Xception (prediction of retinal age)	UK Biobank	1. Retinal age gap (Xception-predicted age minus chronological age): AUC = 0.676 2. Risk-factor model: AUC = 0.661
Zhou et al. (2023)	2023	2,526 (1,263/1,263/0) 308 (154/154/0)	Fundus photography, OCT-A	RETFound (masked autoencoder for SSL, MLP for stroke classification), SL-ImageNet, SSL-ImageNet, SSL-Retinal	MEH-MIDAS, MEH-AlzEye, UK Biobank	RETFound Performance for Stroke Prediction: 1. Internal Dataset (MEH-AlzEye): AUROC = 0.754. 2. External Dataset (UK Biobank): AUROC = 0.559–0.594. 3. Better performance than SL-ImageNet and SSL-Retinal.
Outcome prediction						
Khan et al. (2022)	2022	56 (21/35/0)	OCT	SVM (Classification of pial collateral status)	Hamad General Hospital	1. Retinal Vessel Multifractal Dimensions: Significantly higher in patients with poor pial collaterals compared to good pial collaterals. 2. SVM Model for classification: Accuracy = 0.743 Sensitivity = 0.743 Specificity = 0.707

DMPMelb: Diabetic Management Programme (Melbourne); EPIC-Norfolk: European Prospective Investigation into Cancer and Nutrition - Norfolk Study; MEH-MIDAS: Moorfields Eye Hospital Medical Image Dataset for AI Systems; MCRS: Multi-Centre Retinal Stroke Study; RASTA: Retinal oct-Angiography and cardiovascular STAtus dataset; ROSE: Retinal OCT-Angiography Vessel Segmentation Dataset; SCES: Singapore Chinese Eye Study; SiMES: Singapore Malay Eye Study; SiNDI: Singapore Indian Eye Study; SP2: Singapore Prospective Study Program.

## 7 Challenges and limitations

Investigating retinal biomarkers of stroke encounters several challenges and limitations. The lack of standardized protocols while selecting retinal imaging modalities (such as fundus photography, OCT, and OCTA) for distinguishing individual retinal features makes it difficult to compare findings across studies. Furthermore, the abundance of image acquisition techniques, numerous analytic approaches, and the expression of the results using divergent parameters can lead to inconsistent interpretations of retinal features associated with the risk of stroke (Girach et al., 2024). Furthermore, the precise biological mechanisms linking specific retinal vascular changes to stroke risk are not fully understood and require further elucidation, which could enhance the logical process behind AI-driven modules and lead to higher accuracy.

Current predictive models using retinal imaging have not exhibited consistent and substantially better performance while comparing with traditional risk scores. Although AI algorithms show promise in analyzing retinal images, there are concerns about the accuracy of these predictive models. It has been indicated that while features seen in retinal imaging may suggest the risk of stroke, they do not consistently provide better predictive capabilities than established clinical risk assessment tools. Additionally, the effectiveness of retinal imaging in different populations, across various demographic categories, and clinical settings needs to be confirmed as a tool for assessing stroke risk through more extensive validation studies, and their generalizability constitutes a major concern. However, many existing studies rely on limited groups that may not accurately represent the wider population at risk for stroke.

Developing reliable AI models requires high-quality, diverse datasets with larger populations for training and validation. Besides, annotating retinal images for training AI models requires expert input, limiting the availability of sufficiently large and well-annotated datasets and drawing attention to the widely considered TL algorithms (Zhou et al., 2023; Tan et al., 2024).

The use of AI in analyzing retinal images raises ethical and regulatory concerns. Data privacy, informed consent, and the potential for algorithmic bias need to be addressed to ensure the responsible use of AI applications in retinal imaging. Additionally, the regulatory frameworks governing the use of AI in healthcare are still evolving, which can create uncertainty for clinicians and researchers (Zhou et al., 2023; Tan et al., 2024).

## 8 Future direction

Outlining the direction of future research would become more achievable, acknowledging the current research horizon and broadening it by applying the solutions found to narrow the gaps and resolve the challenges and controversies. In summary, AI models trained on large retinal imaging datasets, along with patient demographics and clinical data, play a key role in accurately identifying stroke risk and predicting future events. These happen through the identification of specific retinal vasculature changes, which are either directly or indirectly associated with heightened stroke risk. Current studies suggest that features such as retinal vessel diameter, tortuosity, and the presence of specific retinal pathologies may serve as indicators of stroke risk. One of the most beneficial and prominently discussed functions of AI algorithms within our topics constitutes the development of stroke risk stratification tools that categorize patients into different risk groups. Additionally, the AI-powered Retinal image analyzers could potentially detect strokes without apparent clinical symptoms, called silent stroke, that may precede major strokes and mandate the clinicians for timely intervention and preventative measures. AI can also be used to track changes in retinal vasculature after stroke treatment, helping to assess treatment efficacy and personalize follow-up care.

Future research in analyzing retinal images for stroke diagnosis and management requires developing more robust and generalizable AI models. This escalation necessitates algorithms with refined capability in handling big data, analyzing more diverse datasets, and employing techniques to enhance model interpretability (using explainable AI (XAI) techniques). They aim to improve the accuracy and reliability of predicting strokes from retinal images, explaining the performed functions and exhibited results. Improving the interpretability of AI models is crucial for gaining clinician trust and facilitating the adoption of these technologies in clinical workflows. Future developments should focus on creating XAI systems that provide insights into the decision-making processes (Mesinovic et al., 2023; Abdollahi et al., 2024; Frasca et al., 2024). By offering transparent explanations of decision-making processes, AI systems can help clinicians validate recommendations and foster a more collaborative approach to patient care.

Moreover, future research would further gain advantage of the capability of the AI models to transfer the determined weights of model training from the source domain to the target domain, also known as TL, which could substantially enhance model efficacy in small datasets. These models can analyze complex patterns of distortion in retinal blood vasculature that may be linked to cerebrovascular conditions (Arnould et al., 2023). By training AI systems on extensive and diverse datasets, researchers can improve the generalizability of these models, confirming their external validity and ensuring their reliability and effectiveness across different populations and clinical settings. This validation process is essential for building trust in the clinical utility of retinal imaging technologies (Pachade et al., 2022; Zhou et al., 2023). Furthermore, combining retinal imaging modalities with the results of other diagnostic methods, such as MRI and blood tests, can provide a more comprehensive stroke risk evaluation, leading to better-informed comprehensive clinical decisions (Chew et al., 2024). The integration of Quantum AI into stroke management holds promise for revolutionizing diagnostics, treatment, and research. However, the full realization of these applications will require ongoing collaboration between AI researchers, clinicians, and healthcare institutions to address the challenges associated with implementation in clinical practice (Zeleňák et al., 2021; Davids et al., 2022; El Naamani et al., 2024).

Future investigations should aim to identify and validate additional biomarkers through longitudinal studies tracking changes in retinal health over time (MacGillivray et al., 2014; Phadikar et al., 2017). These studies are also crucial for enhancing the predictive accuracy of existing AI models (Carrasco-Ribelles et al., 2023). Furthermore, long-term, larger-scale, and prospective studies are crucial in understanding how changes in the retina over time are connected to stroke risk and outcomes (Wolcott and English, 2024). This approach could lead to the development of more robust predictive models that combine traditional risk factors with novel retinal indicators. Future efforts should prioritize the standardization of retinal imaging protocols to address the variability in data quality and interpretation. Establishing clear image acquisition, processing, and analysis guidelines will ensure consistency across studies and clinical applications. Standardization will further facilitate this integration (Lee et al., 2021; Rudnicka et al., 2022; Sampson et al., 2022). Additionally, to improve AI integration in clinical care, the establishment of AI Quality Improvement (AI-QI) units in hospitals has been proposed. These units would use tools like statistical process control to monitor algorithm performance, enhancing stroke patient care and recovery (Feng et al., 2022).

Establishing robust ethical and regulatory frameworks will be essential as AI technologies advance. These frameworks should address concerns related to data privacy, informed consent, and algorithmic bias. Ensuring patient safety and equity in AI model development will be essential for fostering their effective and fair implementation in stroke care (Solanki et al., 2023).

## 9 Conclusion

This research reviews the use of artificial intelligence algorithms in diagnosing and evaluating stroke risk through retinal imaging findings. We demonstrated that artificial intelligence improves stroke diagnosis and risk stratification performance, although further studies are required to confirm the validity and applicability of the obtained results across different datasets and various populations. Novel techniques would guide the researchers through overcoming the challenges of AI applications.
